# RIP1-dependent Apoptosis and Differentiation Regulated by Skp2 and Akt/GSK3β in Acute Myeloid Leukemia

**DOI:** 10.7150/ijms.68385

**Published:** 2022-03-06

**Authors:** Wenran Dan, Liang Zhong, Zhonghui Zhang, Peng Wan, Yang Lu, Xiao Wang, Zhenyan Liu, Xuan Chu, Beizhong Liu

**Affiliations:** 1 Central Laboratory of Yong-Chuan Hospital, Chongqing Medical University, Chongqing 402160, China.; 2 Key Laboratory of Laboratory Medical Diagnostics, Ministry of Education, Department of Laboratory Medicine, Chongqing Medical University, Chongqing 400016, China.

**Keywords:** Skp2, RIP1, GSK3β, Acute myelocytic leukemia

## Abstract

Acute myeloid leukemia (AML) is a heterogeneous neoplasm characterized by variations in cytogenetics and molecular abnormalities, which result in variable response to therapy. Receptor-interacting serine/threonine kinase 1 (RIP1)-mediated necroptosis has been reported to have a potential role in the treatment of AML. We obtained Skp2 and RIP1 are significantly overexpressed in AML samples using original published data, and identified that Skp2-depletion in AML cells significantly suppressed RIP1. Functional analysis showed that the inhibition of RIP1 caused by necrostatin-1 (Nec-1) inhibited the proliferation, simultaneously facilitate both the apoptosis and differentiation of AML cells. Mechanistical analysis elucidated that knockdown of Skp2 suppresses RIP1 by transcriptional regulation but not by proteasome degradation. Additionally, Skp2 regulated the function of RIP1 by decreasing K63-linked ubiquitin interaction with RIP1. Moreover, the suppression of Akt/GSK3β was observed in Skp2 knockdown stable NB4 cells. Also, GSK3β inactivation via small-molecule inhibitor treatment remarkably decreased RIP1 level. RIP1 regulates differentiation by interacting with RARα, increasing RA signaling targets gene C/EBPα and C/EBPβ. In conclusion, our study provides a novel insight into the mechanism of tumorigenesis and the development of AML, for which the Skp2-Akt/GSK3β-RIP1 pathway can be developed as a promising therapeutic target.

## Introduction

Acute myeloid leukemia (AML) is an aggressive malignancy characterized by a clonal disorder of hematopoietic stem and progenitor cells caused by acquired and inherited genetic variation, which results in the accumulation of aberrant proliferation and undifferentiation myeloblasts/promyelocytes 1-3. There is an urgent need for studies on novel therapeutic targets considering the poor prognosis of patients with AML(the worst median overall survival is 2.67 months and nearly 80% will die within one year for patients at age ≥ 65 years) 4. However, limited understanding of the mechanism has restricted the identification of novel molecular targets for AML.

Considering that the majority of the current dominant therapeutic strategies exert their antileukemic effects by triggering programmed cell death, namely, by apoptosis and necroptosis, crosstalk exists between the two pathways. Resistance to treatment has been studied extensively, owing to the inactivation of the apoptotic pathway, as well as engagement necroptosis, which has evolved as a novel therapeutic approach 5-9. Receptor-interacting serine/threonine kinase protein 1 (RIP1) is a key regulator of necroptosis that closely aligns with RIP3. Persistent activation of RIP1/RIP3 signaling has been observed in AML cells, and RIP1/RIP3 knockout attenuates the leukemogenic capacity 10, 11. The role of RIP1 in the interactive molecular pathways of cell differentiation, apoptosis and necroptosis highlights its importance in the occurrence and development of cancer 12-15. However, the mechanisms of signaling pathways activated on regulating RIP1 remain poorly understood. Several studies have reported that Akt kinase activity is critical for necroptosis and is activated in an RIP1-dependent manner 16-18, which revealed the interaction between Akt activation and RIP1 activity during necroptosis.

In this study, we aimed to unveil that RIP1 regulated by and regulating Akt/GSK3β pathway in the development of AML. We discovered the Skp2-Akt/GSK3β axis is important for RIP1 expression. Moreover, we found that RIP1 could interaction with RARα to regulate differentiation in AML. Altogether, our results showed that RIP1 has oncogenic function depend on Skp2-Akt/GSK3β axis and the inhibition of RIP1 is required for AML differentiation.

## Materials and methods

### Microarray data analysis

Public microarray data of Skp2 and RIP1 mRNA expression in the bone marrow (BM) and peripheral blood of AML patients and healthy donors were obtained from the National Center for Biotechnology Information (NCBI) Gene Expression Omnibus (GEO) database. Transcriptome sequencing data of 26 AML patients and 10 normal peripheral blood samples were selected from GSE9476. Transcriptome sequencing data of 96 AML patients were obtained from GSE21261.

### Cell culture and lentivirus infection

Human AML NB4, U937, THP-1 and KG1α cell lines were purchased from ATCC and cultured in RPMI-1640 (Gibco-Life Technologies, Carlsbad, CA, USA) containing 10% fetal bovine serum (FBS; Gibco, Melbourne, Australia) and 1% penicillin/streptomycin (Beyotime, Shanghai, China). Stable shSkp2-expressing NB4 and U937 cells were established by transduction with U6-Puro-shSkp2-derived lentivirus (Genechem, Shanghai, China). After 15 d of transfection, puromycin (Sigma, St Louis, MO, USA) containing 4 mg/mL was cultured cells for 14 d. The cultured cells were placed in a 37 °C sterile incubator containing 5% CO_2_.

### Small interference RNA (siRNA) and transfection

The siRNA constructs for the Skp2 and control groups were obtained from Sangon Biotech (Shanghai, China) and transfected with Lipofectamine 2000 (Invitrogen Life Technologies, CA, USA) according to the manufacturer's instructions. The cells were collected for the experiments after 24h or 48h.

### Reagents and antibodies

Cycloheximide (CHX; MCE, USA) at a concentration of 25 µg/mL, MG132 (MCE,USA) at a concentration of 5 µg/mL, SB216763 (MCE, USA) at a concentration of 10 μM, ATRA (Sigma, St Louis, MO, USA) at a concentration of 1 μM and necrostatin-1 (MCE, USA) at a concentration of 30 μM were added to treat cells.

The following specific primary antibodies were used: anti-RIP1 (Cell Signaling Technology, #73271), anti-Skp2 (Santa Cruze Biotechnology, sc-74477), anti-p53 (Cell Signaling Technology, #2527), anti-p90 (Cell Signaling Technology, #9355), anti-SOX2 (Cell Signaling Technology, #3579), anti-Akt (Abcam, ab17463), anti-GSK3β (Cell Signaling Technology, #12456), anti-phospho^ser 9^-GSK3β (Cell Signaling Technology, #5558), anti-Bcl2 (Abcam, ab32121), anti- ubiquitin (Santa Cruze Biotechnology, sc-8017), anti-K63-linkage specific polyubiquitin (Cell Signaling Technology, #5621),anti-K48-linkage specific polyubiquitin (Cell Signaling Technology, #8081), anti-c-Myc (Abcam, ab32072), anti-RARα (Santa Cruze Biotechnology, sc-515796), anti-C/EBPα (Cell Signaling Technology, #8178), anti-C/EBPβ (Cell Signaling Technology, #3082), anti-H3 (Bioss, no.33942M), anti-GAPDH (Cell Signaling Technology, #5174), and anti-β-actin (Boster, no.BM0627).

### Cell viability assay

The cells were seeded in 96-well plates and were subjected to different treatment. The measurements were recorded every 12h for three consecutive days based on the absorbance of the sample at 450 nm by using Cell Counting Kit 8 (CCK-8; MCE, USA).

### Western blot analysis

Tumor cells were lysed with RIPA buffer containing 1% PMSF for 30 min, and the protein content of which was determined by using the BCA protein assay kit (Beyotime, Shanghai, China). After SDS electrophoresis and transfer, the PVDF membranes were blocked with 5% milk powder in TBST for 2h at room temperature, followed by overnight incubated with primary antibodies at 4 °C overnight. After incubation with horseradish peroxidase linked IgG, the immunoreactivity was visualized by chemiluminescent substrate (ECL) and the protein signal was detected using the Bio-Rad imaging system (Hercules, CA, USA).

### Co-immunoprecipitation (co-IP)

Cells were collected and lysed using NP40 containing 1% PMSF. Equal amounts of protein were incubated overnight with protein A/G magnetic beads (MCE, USA) and anti-RARα antibody at 4℃. The beads were washed thrice with PBST, and subsequently boiled in 2× SDS loading buffer for further electrophoresis.

### Real-time reverse transcription quantitative polymerase chain reaction (RT-qPCR)

Total RNA was extracted from the cells using TRIzol (Takara Biotechnology, Dalian, China) and reverse transcribed into cDNA using the PrimeScript^RT^ Reagent Kit (Takara Biotechnology, Dalian, China) according to the manufacturer's instructions. Real-time reverse transcription quantitative polymerase chain reaction (RT-qPCR) was performed using SYBR Green (Takara Biotechnology, Dalian, China). β-actin was used as the internal control. The primer sequences used in the experiments are as following:

- RIP1 5'-TTACATGGAAAAGGCGTGATACA-3' (forward) and 5'-AGGTCTGCGATCTTAATGTGGA-3' (reverse);
- CD11b 5'-CAGAGCGTGGTCCAGCTTCA-3' (forward) and 5'-CCTTCATCCGCCGAAAGTCA-3' (reverse),
- CD14 5'-AAGCACTTCCAGAGCCTGTC-3' (forward) and 5'-TCGTCCAGCTCACAAGGTTC-3' (reverse),
- C/EBPβ 5'-ATGTTCCTACGGGCTTGTTG-3' (forward) and 5'-CCCAAAGGCTTTGTAACCA-3' (reverse),
- β-actin 5'-TGACGTGGACATCCGCAAAG-3' (forward) and 5'-CTGGAAGGTGGACAGCGAGG-3' (reverse).

### Flow cytometric analysis

Staining of apoptotic cells was performed with the Annexin V-PI Apoptosis Detection Kit (BD Biosciences, CA, USA) according to the manufacturers' instructions. For the cell cycle analysis, following treatment with Nec-1 for 48h, the cells were washed in PBS and fixed in 70% ethanol overnight at -20 °C. Following this the cells were incubated with PI and RNase for 30 min at 37 °C after being washed with PBS. Before flow cytometry, the cells were stained with the CD11b-PE antibody (BioLegend, no. 301306) for the differentiation analysis.

### Statistical analysis

Statistical analyses were performed using Prism (GraphPad). Data are presented as mean ± SD, and the unpaired Student's t -test and ANOVA were used to calculate the p-value. P<0.05 was considered statistically significant.

## Results

### Skp2 and RIP1 expression in AML patients

**Figure 1:**
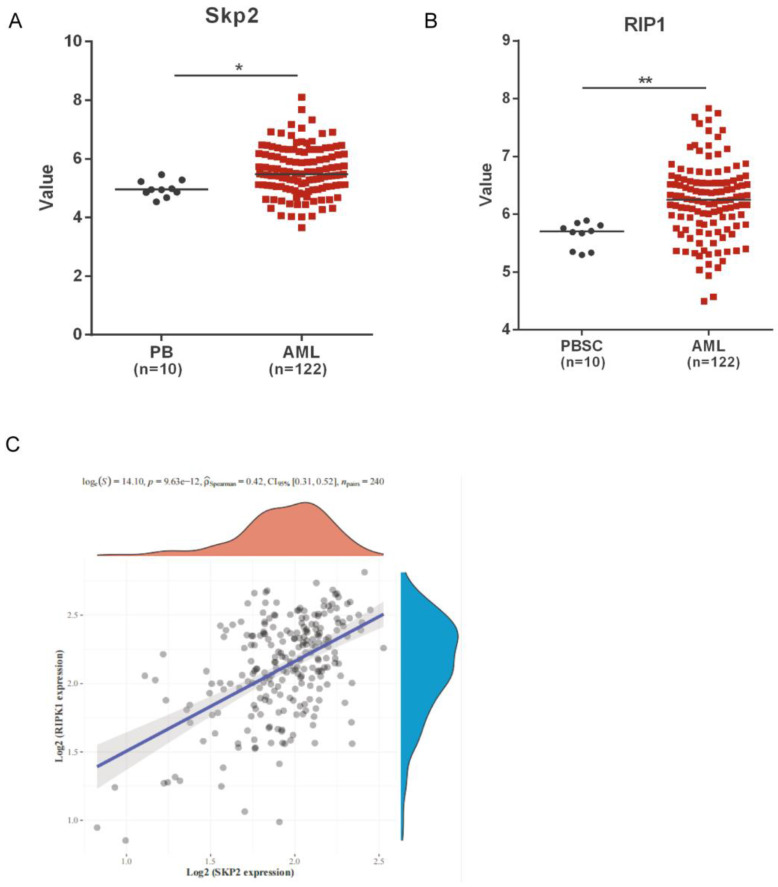
** Skp2 and RIP1 expression in AML patients. (A-B)** Analysis of Skp2 and RIP1 mRNA expression in AML BM and normal peripheral blood cells; datasets were obtained from the GEO database (PB: peripheral blood; PBSC: peripheral blood stem cells, CD34^+^). **(C)** The Skp2 and RIP1 mRNA expression in AML BM were download from TARGET, We used Spearman's correlation analysis to describe the correlation between Skp2 and RIP1. A p-value of less than 0.05 was considered statistically significant. Data analysed by Student's t-test,*p< 0.05, **p< 0.01.

We analyzed the public microarray data to determine the role of Skp2 and RIP1 in AML. The data revealed a marked upregulation in the expression of both Skp2 and RIP1, in AML patients compared to that in healthy donors (Fig. 1A-B). Raw counts of RNA-sequencing data of Skp2 and RIP1 were obtained from Therapeutically Applicable Research To Generate Effective Treatments (TARGET) dataset (https://ocg.cancer.gov/programs/target), Spearman correlation analysis of Skp2 gene expression and RIP1 expression was performed and the data shown that the positive correlation was presented between the two gene in AML (Fig. 1C). The results revealed that Skp2 and RIP1 are highly expressed in AML, might jointly participate in the development of AML.

### Skp2 knockdown promotes apoptosis and inhibits proliferation of AML cells *via* inhibition of RIP1

**Figure 2:**
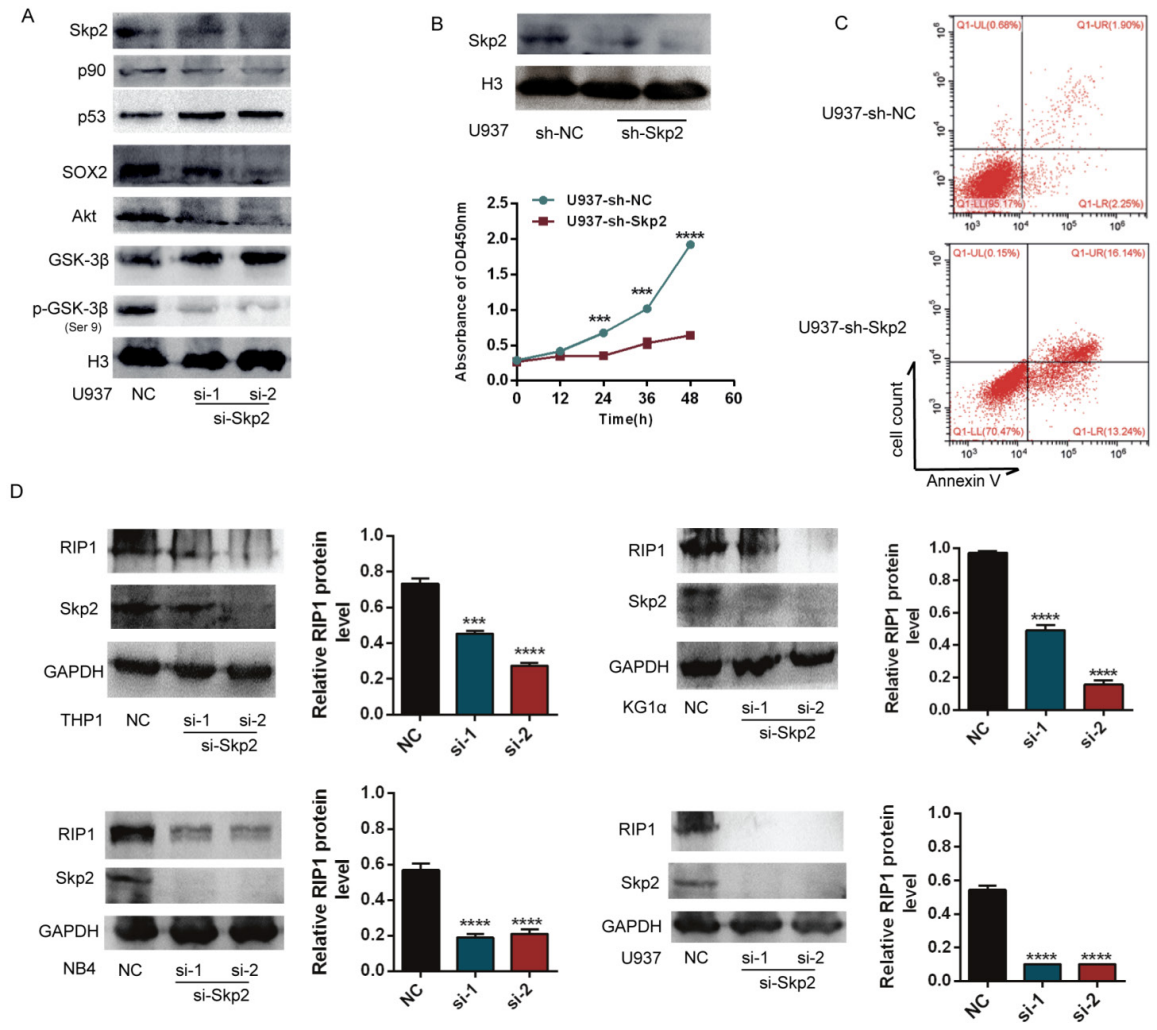
** Skp2 knockdown promotes apoptosis and inhibits proliferation of AML cells *via* inhibition of RIP1. (A)** Successful knockdown of Skp2 in U937 cells. The expression levels of p53 and GSK3β were increased, whereas those of p90, SOX2, Akt and p^Ser 9^-GSK3β, were decreased. **(B)** Attenuation of U937 cell proliferation ability upon Skp2 knockdown was detected by CCK-8. **(C)** Flow cytometry analysis of annexin V-PI-stained U937 cells with successful Skp2 depletion. **(D)** Lysates from THP1, KG1α, NB4, and U937 cells transfected with control or Skp2 siRNA for 48h were subjected to Western blot analysis for the expression of RIP1, Skp2, and GAPDH (loading control). Data are presented as the mean ± SD of three independent experiments and analyzed by Student's t-test, ***p< 0.001, ****p<0.0001.

To analyse the potential handicap for myeloid malignant cells caused by decreased Skp2 expression, the expression of Skp2 was downregulated in U937 cells using specific siRNAs to confirm its effects on AML cells. Western blotting showed that the downregulation of Skp2 increased the expression levels of p53, but decreased that of p90, SOX2, which are proliferation-associated proteins. The inhibition of Akt/GSK3β signaling was also confirmed upon Skp2 knockdown (Fig. 2A). Subsequently, the effects of Skp2 on U937 cell proliferation and survival were determined using the CCK-8 and FACS (Fig. 2B-C). The results revealed that cell proliferation was inhibited and apoptosis was promoted upon Skp2 knockdown. Considering that RIP1 has been reported to possess the capacity of promoting AML. We further investigated whether Skp2 knockdown with specific siRNA affects the expression of RIP1. As we expected, immunoblotting experiments revealed that Skp2 depletion led to the inhibition of RIP1 in AML cells (Fig. 2D).

### Inhibition of RIP1 protein suppresses cell proliferation in Skp2-depleted AML cells

**Figure 3:**
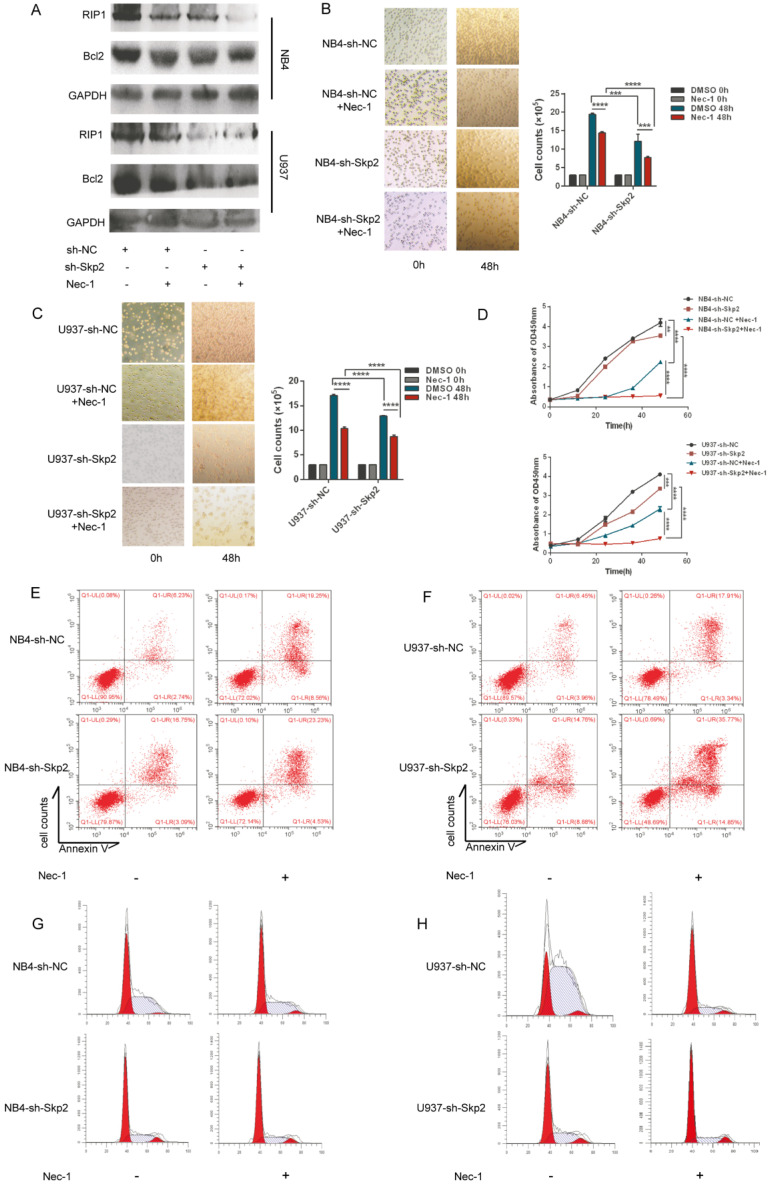
** Inhibition of RIP1 protein suppresses cell proliferation in Skp2-depleted AML cells. (A-D)** NB4 and U937 cells were subjected to Skp2 knockdown and treated with necrostatin-1 (Nec-1, 30 µM) for 48h; apoptosis determined via immunoblotting (A). Proliferation determined by cell counting (B-C) and CCK-8 (two-way ANOVA, p<0.05 considered to be statistically) (D). **(E-F)** Inhibition of RIP1 by treatment with Nec-1 led to a marked increase in Skp2 depletion-induced apoptosis detected *via* flow cytometry. **(G-H)** Downregulation of Skp2 led to a marked arrest in cell cycle progression *via* inhibition of RIP1, as characterized by an accumulation of NB4 and U937 cells in the G0/G1phase. Data presented as the mean ± SD of three independent experiments and analyzed by Student's t-test, **p< 0.01, ***p< 0.001.

RIP1 is an important mediator of cell death. We investigated whether kinase activity was involved in promoting proliferation of AML cells, considering that the expression of RIP1 is regulated by Skp2. NB4 and U937 cells, with or without Skp2 depletion, were treatment with the RIP1 kinase inhibitor, Nec-1, which facilitated knockdown of Skp2-induced apoptosis (Fig. 3A), suggesting that RIP1 lacking kinase activity induced apoptosis both in NB4 and U937 cells. To further analyze the facilitation for myeloid cells proliferation due to RIP1 activity, we used Nec-1 and observed that both NB4 and U937 cells were inhibited cell viability, and simultaneously enhanced the inhibitory effect of Skp2 knockdown on cell proliferation (Fig. 3B-C). Similar results were confirmed through CCK8 that the proliferation inhibition mediated by Skp2-knockdown was nearly totally enhanced by Nec-1 (Fig. 3D). In addition, the flow cytometry results revealed that inhibition of RIP1 activity promoted apoptosis (Fig. 3E-F) and cycle arrest in the G0/G1 phase (Fig. 3G-H) induced by Skp2 knockdown both in NB4 and U937 cells. Together, our results indicate that RIP1 kinase may play an important role in Skp2-induced proliferation and cell cycle arrest.

### Skp2 regulates the expression and function of RIP1 through transcriptional regulation and K63-linked ubiquitination

**Figure 4:**
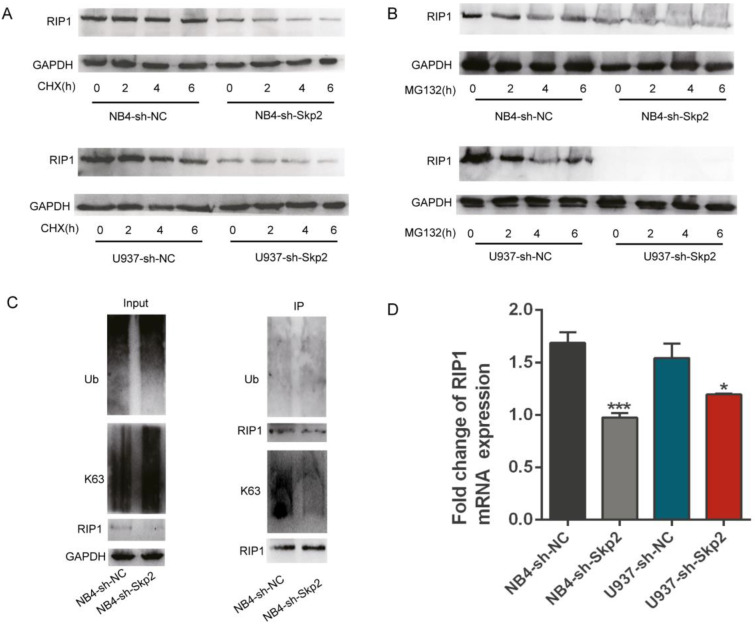
** Skp2 regulates the expression and function of RIP1 through transcriptional regulation and K63-linked ubiquitination. (A)** Cells were treated with 25 µM CHX for 2h, 4h and 6h to analyzed the stability of RIP1 regulated by Skp2; RIP1 protein levels were detected by Western blotting. **(B)** Cells were treated with 5 µM MG132 for 2h,4h and 6h to determine whether RIP1 was degraded via the ubiquitin-proteasome system (UPS) regulated by Skp2. Western blotting was performed to evaluate the protein levels of RIP1. **(C)** Co-immunoprecipitation analysis showing a reduction in K63-linked ubiquitination but no significant total ubiquitination of RIP1 upon Skp2 knockdown. **(D)** RT-qPCR analysis showing that RIP1 mRNA reduction in NB4 and U937 cells upon Skp2 knockdown. Data represented the mean ± SD of three independent experiments and analyzed by Student's t-test, *p< 0.05, **p< 0.01, ***p< 0.001.

Ubiquitination of RIP kinases is one of the most important post-translational modifications known to dictate the fate of cells 19-23. As described before, Skp2 is an essential E3 ligase that regulates cell death and other processes by modifying the ubiquitination status of their substrate proteins 24, 25. In order to verify whether Skp2 has the function on ubiquitination of RIP1, we performed the CHX chase assay to assess the effect of Skp2 knockdown on the endogenous RIP1 protein to identify whether Skp2 regulated the expression of RIP1 via the ubiquitin-proteasome system (UPS). As shown in Fig. 4A, Skp2 knockdown decreased the expression of RIP1, however, it did not have any significantly effect on the stability of RIP1 protein, ruling out the possibility that the decreased RIP1 expression could result from enhanced the ubiquitination rate of RIP1. Furthermore, endogenous Skp2 depletion by MG132 treatment significantly decreased RIP1 expression (Fig. 4B). MG132, a protease inhibitor as well as an inducer of apoptosis, indicated that the regulation of RIP1 by Skp2 is not via the UPS, and that RIP1 contributes to the Skp2-regulated apoptotic pathway. Additionally, knockdown of Skp2 expression has no significant of total ubiquitin on RIP1; however, it decreased the K63-linked polyubiquitin chains on RIP1 in NB4 cells (Fig. 4C). Thus, Skp2 is required for K63-linked polyubiquitination of RIP1, Indicating that the mechanism of the function of RIP1 driven by Skp2 was dependent on the K63-linked post-translational modification of RIP1.This observation is consistent with the previously reported finding that K63-associated de-ubiquitination of RIP1 could trigger apoptosis 26, 27. Furthermore, we observed that deregulation in RIP1 mRNA expression in NB4 and U937 cells upon Skp2 depletion (Fig. 4D). Further confirmed that the expression of RIP1 regulated Skp2 was not dependent on proteasome degradation but through transactivation. Taken together, our data indicated that knockdown Skp2 decreased RIP1 expression by suppressing transcription and inhibited RIP1 function by reducing its K63-linked ubiquitination.

### Inhibition of the RIP1 protein negatively regulates the Akt/GSK3β pathway and GSK3β dephosphorylation suppresses RIP1 expression

**Figure 5:**
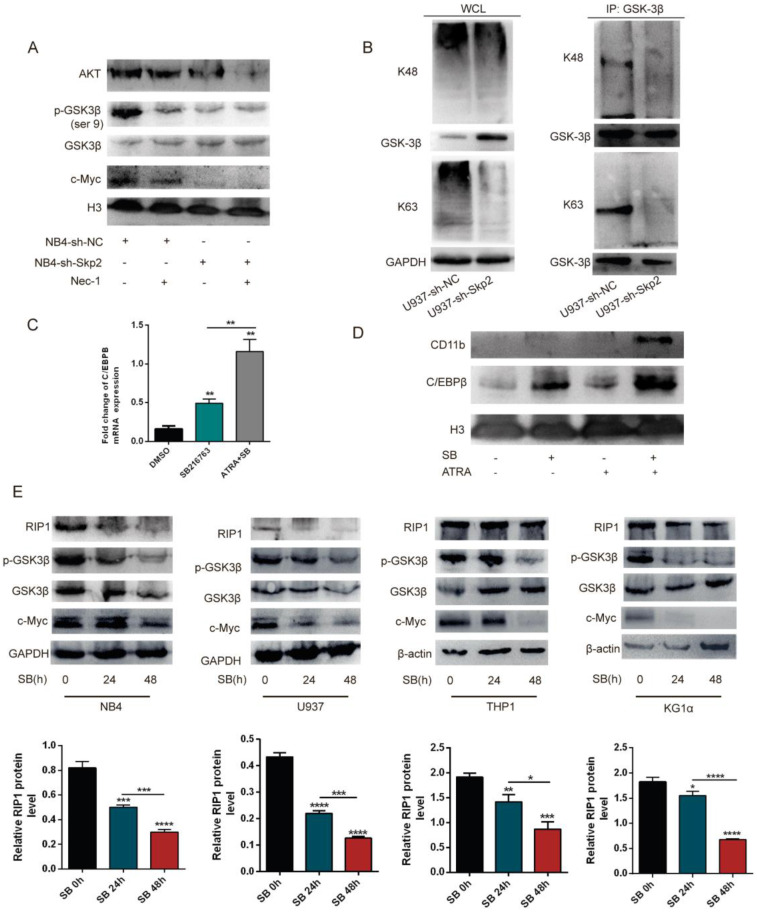
** Inhibition of the RIP1 protein negatively regulates the Akt/GSK3β pathway and GSK3β dephosphorylation suppresses RIP1 expression. (A)** NB4 cells with control or Skp2 knockdown, treated with or without Nec-1, harvested after 48h, followed by Western blot analysis. **(B)** An endogenous IP assay demonstrating that the knockdown of Skp2 resulted in a reduction of GSK3β in both K48- and K63-linked polyubiquitination of U937 cells. **(C)** C/EBPβ mRNA levels in U937 cells treated with SB216763 alone or in combination with ATRA (10^-6^ M) determined by RT-qPCR. **(D)** The effects of the GSK3β activation concerning ATRA-induced differentiation in U937 cells. U937 cells were treated with ATRA, SB216763, or a combination of these agents. Cell extracts were prepared and the levels of the indicated proteins were detected by immunoblotting. **(E)** Immunoblotting and quantification of protein levels upon treatment with SB216763 (GSK3β inhibitor) at the indicated times in various AML cell lines. Data presented as the mean ±SD of three independent experiments. P values were determined by Student's t-test. *p< 0.05, **p< 0.01, ***p< 0.001.

To elucidate the molecular mechanisms underlying the Skp2 regulated RIP1 involved in the cell apoptosis, the Akt/GSK3β pathway was detected by WB for their critical role in Nec-1 induced apoptosis. The results showed that treatment of NB4 cells with Nec-1, with or without Skp2 depletion, further inhibited the expression of Akt and c-Myc as well as the phosphorylation of GSK3β, which is the downstream kinase of the PI3K/Akt pathway (Fig. 5A). Additionally, the co-IP experiment was performed to verify the role of Skp2 in the functional regulation of GSK3β. The results revealed that the knockdown of Skp2 reduced both K48- and K63-linked polyubiquitination of GSK3β in U937 cells (Fig. 5B). To identify the growth regulatory ability of GSK3β in AML cells, we demonstrated that SB216763 (GSK3β inhibitor) increased the protein levels and mRNA levels of C/EBPβ (Fig. 5C), as well as C/EBPβ and CD11b were further increased following SB combination with ATRA in U937 cells (Fig. 5D). These data implied that targeting Skp2 prevented the growth of AML cells in a GSK3β-mediated manner.

Although GSK3β is directly regulated by Skp2, here we determined whether the activity of GSK3β can influence the expression of RIP1, SB216763 was used to inhibit the activation of GSK3β in AML cell lines to validate whether Skp2 regulates RIP1 through the level of p-GSK3β. Of note, the decreased activation of GSK3β in the NB4, U937, THP1 and KG1α cells led to the reduction in RIP1 expression, indicating that the inhibition of GSK3β activity plays a role in the regulation of RIP1 (Fig. 5E). These results suggest that Skp2-regulated RIP1 levels critically depend on the Akt/GSK3β signaling for AML cell survival.

### Inhibition of RIP1 promotes the differentiation of AML cells but RIP1 is required for ATRA-induced differentiation

**Figure 6:**
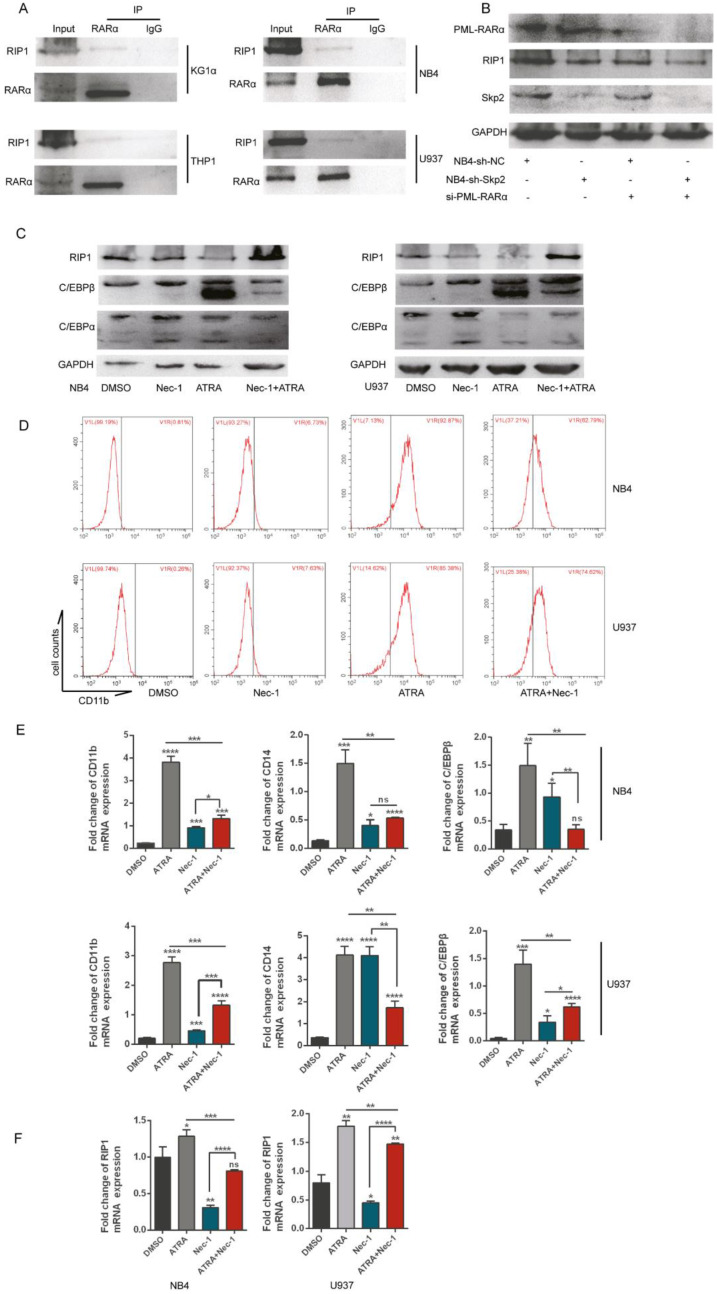
** Inhibition of RIP1 promotes the differentiation of AML cells but RIP1 is required for ATRA-induced differentiation. (A)** Co-immunoprecipitation analysis showing the physical interaction between endogenous RIP1 and RARα proteins in AML cells. **(B)** NB4-sh-NC and NB4-sh-Skp2 cells were transfected with PML-RARα siRNA respectively, following harvested the cells for Western blot analysis. **(C-D)** NB4 and U937 cells were treated with Nec-1, ATRA, and a combination of the two agents for 48h, followed by immunoblotting analysis (C) and collected to analyzed CD11b-positive cells by flow cytometry assay (D). **(E)** RT-qPCR experiments determined the quantification mRNA levels of CD11b, CD14 and C/EBPβ following treatment with Nec-1, ATRA, and Nec-1 plus ATRA for 24h in NB4 and U937 cells. **(F)** RT-qPCR experiments determined the quantification mRNA levels of RIP1 following treatment with Nec-1, ATRA, and Nec-1 plus ATRA for 24h in NB4 and U937 cells. Data presented as the mean ± SD of three independent experiments. P values were determined by Student's t-test. *p< 0.05, **p< 0.01, ***p< 0.001.

Further studies determined whether RIP1 played important role in AML cells differentiation, we performed the co-IP experiment in various AML cell lines to analyze the role of RIP1 in the differentiation of AML cells, which indicated that RARα could interact with RIP1 (Fig. 6A). RARα is a specific differentiation marker required for the granulopoiesis 28, 29. We performed immunoblotting to determine the change in the expression of RIP1 upon PML-RARα knockdown with si-RNA in NB4 cells, with or without Skp2 depletion, to gain insights into the correlation between RIP1 and RARα. Downregulation of PML-RARα further attenuated the decreased expression of RIP1 in Skp2-knockdown NB4 cells (Fig. 6B). Next, we analyzed the expression levels of RA-specific target genes, C/EBPβ and C/EBPα (C/EBPβ target gene) 30, 31, whose expressions are controlled by RARs and a RARE in the respective promoter regions of these genes. Immunoblotting revealed that C/EBPα and C/EBPβ were dramatically elevated in NB4 and U937 cells treated with Nec-1; however, RIP1 kinase inactivation reduced the levels of ATRA-induced C/EBPα and C/EBPβ (Fig. 6C). While it was observed that NB4 and U937 cells treated with Nec-1 promoted expression of CD11b, ATRA-induced differentiation was found to be suppressed (Fig. 6D). These observations support the involvement of RIP1 in RA signaling to induce the differentiation of AML cells. The RT-qPCR analysis further confirmed that Nec-1 treatment induces the expression of CD11b, CD14 and C/EBPβ (Fig. 6E), and reduces the ATRA-induced levels of these RA signaling target genes. In addition, we observed that ATRA could increase the RIP1 mRNA level but was reduced by Nec-1 in NB4 and U937 cells (Fig. 6F). Our results indicate that RIP1 might play an important role in ATRA-induced differentiation NB4 and U937 cells. Furthermore, the differentiation of AML cells could be increased by decreasing RIP1 alone, which supporting the hypothesis that the abnormal expression and dysfunction of RIP1 may be pro- or anti-oncogenic, depending on the genetic background of the cells and microenvironment of the tumor.

## Discussion

Despite the diverse functions of RIP1 in mediating cell survival and death signaling, its involvement in apoptosis in the pathogenesis of cancer is being reported increasingly 32, 33. However, the function and regulatory mechanisms of RIP1 have not been characterized fully in AML. The present study reveals that depletion of Skp2 could attenuate the development of AML through reduced activation of RIP1, accomplished by inhibiting the Akt/GSK3β signaling pathway. Furthermore, inhibition of RIP1 kinase activity resulted in the inactivation of the Akt/GSK3β signaling pathway. These findings characterized the Skp2-induced RIP1-Akt/GSK3β-RIP1 loop that promotes the development of AML, and targeting this axis could be a potential therapeutic strategy.

The applicability of necroptosis in cancer treatment remains controversial because its benefits associated with tumor elimination appear limited 12. However, inhibition of necroptosis may sensitize AML cells and induce differentiation 10. In addition, the current study demonstrated the synergistic repressive effects of Nec-1 and Skp2 depletion, in AML cell lines, thereby suggesting an effective strategy for the treatment of AML. We identified a novel Skp2 target molecule, RIP1, which plays an essential role in cell proliferation, differentiation, and apoptosis. First, we detected a decrease in the expression of RIP1 regulated by Skp2 depletion, therefore, we believe that the mechanism of Skp2-regulated proliferation and differentiation in AML cells is associated with the high levels of RIP1. However, the precise molecular mechanisms of how Skp2 regulates RIP1 and the role of RIP1 in the process of AML remain unclear. We observed that inhibition of RIP1 expression by knockdown of Skp2 appeared to require dephosphorylation of GSK3β. Consistent with previous studies, RIP1 activates the PI3K/Akt pathway to regulate the survival of cells 16-18. We observed that Skp2-knockdown resulted in the inhibition of Akt/GSK3β, which following decreased expression of RIP1, negatively activated Akt/GSK3β to constitute a feedback loop. In addition, our study elaborated that decreased Skp2 expression, reduced the K63-ubiquitinated form of RIP1. As previously described, the post-translational modification of RIP1 significantly regulates its function 26, 27, 34. These results confirm our hypothesis that Skp2 regulated AML by inhibiting the function and expression of RIP1.

Next, we established that RIP1 could bind to RARα; therefore RIP1 might have a potential function in RA signaling. RA signaling targets genes involved in various cellular processes, such as proliferation, differentiation, and apoptosis. ATRA is essential for the development of the hematopoietic system by binding to the RARα receptor to activate RA signaling 35-37. A typical case of RA signaling inhibition resulting in a differentiation blockade is t(15;17), generating a fusion protein (PML-RARα), which is considered a transcription repressor of RARα target genes. However, a pharmacologic dose of ATRA could attenuate the blockade 29. Our study defines a function for RIP1 as a negative regulator of RA target genes and differentiation toward granulocyte; however, the enhancement of RIP1 appears essential for ATRA-induced differentiation. RARα is one of the most important effector during RA signaling activated, the interaction between RIP1 and RARα indicate that RIP1 suppressed AML cell differentiation may through RA signaling. It was recently shown that ATRA could induce cell differentiation and necroptosis dependent on elevated RIP1and RIP3, consistent with our study, RIP1 may act as regulatory targets of ATRA in differentiation to necroptosis transition. Although several pathway have been reported as the molecular mechanisms in differentiation of AML cells, the role of necroptosis is still unclear in the process of differentiation. In this study, we discover that inhibition of necroptosis promoted cell differentiation, and further inhibition of necroptosis inhibited differentiation in ATRA-induced differentiation, suggesting that necroptosis has the ability to pro- and anti- tumor in AML. Our results demonstrate that necroptosis is responsible for ATRA induced differentiation, and the expression level of RIP1 is a sensor of necroptosis and differentiation transformation. These findings indicate that decreased expression of RIP1 was responsible for the differentiation of AML cells; however, RA-induced differentiation was suppressed due to excess inhibition. The mechanism that controls the enhanced expression of RIP1 in RA signaling should be clarified subsequently.

Collectively, our study provides evidence that RIP1 participates in the proliferation and death of AML cells by activating the Akt/GSK3β and RA signaling pathways, and Skp2 regulates this process. These data contribute to understanding the regulatory network in AML and may aid in the development of effective therapeutic strategies for AML.

## References

[1] Pelcovits A, Niroula R (2020). Acute Myeloid Leukemia: A Review. R I Med J (2013).

[2] Garg M, Nagata Y, Kanojia D (2015). Profiling of somatic mutations in acute myeloid leukemia with FLT3-ITD at diagnosis and relapse. Blood.

[3] Kirtonia A, Pandya G, Sethi G (2020). A comprehensive review of genetic alterations and molecular targeted therapies for the implementation of personalized medicine in acute myeloid leukemia. J Mol Med (Berl).

[4] Shallis RM, Wang R, Davidoff A (2019). Epidemiology of acute myeloid leukemia: Recent progress and enduring challenges. Blood Rev.

[5] Rahmani M, Nkwocha J, Hawkins E (2018). Cotargeting BCL-2 and PI3K Induces BAX-Dependent Mitochondrial Apoptosis in AML Cells. Cancer Res.

[6] Davidovich P, Kearney CJ, Martin SJ (2014). Inflammatory outcomes of apoptosis, necrosis and necroptosis. Biol Chem.

[7] Lagadinou ED, Sach A, Callahan K (2013). BCL-2 inhibition targets oxidative phosphorylation and selectively eradicates quiescent human leukemia stem cells. Cell Stem Cell.

[8] Su Z, Yang Z, Xie L (2016). Cancer therapy in the necroptosis era. Cell Death Differ.

[9] Feldmann F, Schenk B, Martens S (2017). Sorafenib inhibits therapeutic induction of necroptosis in acute leukemia cells. Oncotarget.

[10] Xin J, You D, Breslin P (2017). Sensitizing acute myeloid leukemia cells to induced differentiation by inhibiting the RIP1/RIP3 pathway. Leukemia.

[11] Liu Y, Liu T, Lei T (2019). RIP1/RIP3-regulated necroptosis as a target for multifaceted disease therapy (Review). Int J Mol Med.

[12] Gong Y, Fan Z, Luo G (2019). The role of necroptosis in cancer biology and therapy. Mol Cancer.

[13] Seifert L, Werba G, Tiwari S (2016). The necrosome promotes pancreatic oncogenesis via CXCL1 and Mincle-induced immune suppression. Nature.

[14] Wang W, Marinis JM, Beal AM (2018). RIP1 Kinase Drives Macrophage-Mediated Adaptive Immune Tolerance in Pancreatic Cancer. Cancer Cell.

[15] Hanggi K, Vasilikos L, Valls AF (2017). RIPK1/RIPK3 promotes vascular permeability to allow tumor cell extravasation independent of its necroptotic function. Cell Death Dis.

[16] Wu Y-T, Tan H-L, Huang Q (2009). Activation of the PI3K-Akt-mTOR signaling pathway promotes necrotic cell death via suppression of autophagy. Autophagy.

[17] McNamara CR, Ahuja R, Osafo-Addo AD (2013). Akt Regulates TNFalpha synthesis downstream of RIP1 kinase activation during necroptosis. PLoS One.

[18] Park S, Zhao D, Hatanpaa KJ (2009). RIP1 activates PI3K-Akt via a dual mechanism involving NF-kappaB-mediated inhibition of the mTOR-S6K-IRS1 negative feedback loop and down-regulation of PTEN. Cancer Res.

[19] Chen Z, Lin CX, Song B (2020). Spermidine activates RIP1 deubiquitination to inhibit TNF-alpha-induced NF-kappaB/p65 signaling pathway in osteoarthritis. Cell Death Dis.

[20] Moquin DM, McQuade T, Chan FK (2013). CYLD deubiquitinates RIP1 in the TNFalpha-induced necrosome to facilitate kinase activation and programmed necrosis. PLoS One.

[21] de Almagro MC, Goncharov T, Izrael-Tomasevic A (2017). Coordinated ubiquitination and phosphorylation of RIP1 regulates necroptotic cell death. Cell Death Differ.

[22] de Almagro MC, Goncharov T, Newton K, Vucic D (2015). Cellular IAP proteins and LUBAC differentially regulate necrosome-associated RIP1 ubiquitination. Cell Death Dis.

[23] Witt A, Vucic D (2017). Diverse ubiquitin linkages regulate RIP kinases-mediated inflammatory and cell death signaling. Cell Death Differ.

[24] Asmamaw MD, Liu Y, Zheng YC (2020). Skp2 in the ubiquitin-proteasome system: A comprehensive review. Med Res Rev.

[25] Cai Z, Moten A, Peng D (2020). The Skp2 Pathway: A Critical Target for Cancer Therapy. Semin Cancer Biol.

[26] Liu L, Wei Z, Fang R (2020). Giardia duodenalis induces extrinsic pathway of apoptosis in intestinal epithelial cells through activation of TNFR1 and K63 de-ubiquitination of RIP1 *in vitro*. Microb Pathog.

[27] Li B, Cong M, Zhu Y (2017). Indole-3-Carbinol Induces Apoptosis of Hepatic Stellate Cells through K63 De-Ubiquitination of RIP1 in Rats. Cell Physiol Biochem.

[28] Xu A, Zhang N, Cao J (2020). Post-translational modification of retinoic acid receptor alpha and its roles in tumor cell differentiation. Biochem Pharmacol.

[29] De Braekeleer E, Douet-Guilbert N, De Braekeleer M (2014). RARA fusion genes in acute promyelocytic leukemia: a review. Expert Rev Hematol.

[30] Wiper-Bergeron N, St-Louis C, Lee JM (2007). CCAAT/Enhancer binding protein beta abrogates retinoic acid-induced osteoblast differentiation via repression of Runx2 transcription. Mol Endocrinol.

[31] Schwarz EJ, Reginato MJ, Shao D (1997). Retinoic acid blocks adipogenesis by inhibiting C/EBPbeta-mediated transcription. Mol Cell Biol.

[32] Steinhart L, Belz K, Fulda S (2013). Smac mimetic and demethylating agents synergistically trigger cell death in acute myeloid leukemia cells and overcome apoptosis resistance by inducing necroptosis. Cell Death Dis.

[33] Dondelinger Y, Aguileta MA, Goossens V (2013). RIPK3 contributes to TNFR1-mediated RIPK1 kinase-dependent apoptosis in conditions of cIAP1/2 depletion or TAK1 kinase inhibition. Cell Death Differ.

[34] Song YK, Hu BC, Xu L (2019). Productive transcription of miR-124-3p by RelA and RNA polymerase II directs RIP1 ubiquitination-dependent apoptosis resistance during hypoxia. Exp Cell Res.

[35] Martino OD, Welch JS (2019). Retinoic Acid Receptors in Acute Myeloid Leukemia Therapy. Cancers (Basel).

[36] Samarut E, Rochette-Egly C (2012). Nuclear retinoic acid receptors: conductors of the retinoic acid symphony during development. Mol Cell Endocrinol.

[37] Hunsu VO, Facey COB, Fields JZ, Boman BM (2021). Retinoids as Chemo-Preventive and Molecular-Targeted Anti-Cancer Therapies. Int J Mol Sci.

